# Insights into Growing Teratoma Syndrome: An In-Depth Retrospective Analysis of Clinical Features and Treatment Outcomes

**DOI:** 10.1007/s13193-025-02451-0

**Published:** 2025-11-06

**Authors:** Pabashi Poddar, Amita Maheshwari, T. S. Shylasree, Rohini Kulkarni, Biswajit Dash, Jaya Ghosh, Kedar Deodhar

**Affiliations:** 1 Department of Gynaecological Oncology, Gynaecology Oncology Disease Management Group, Tata Memorial Hospital, Homi Bhabha National Institute, Dr Ernst Borges Marg, Parel, Mumbai, 400012 Maharashtra India; 2 Department of Medical Oncology, Gynaecology Oncology Disease Management Group, Tata Memorial Hospital, Homi Bhabha National Institute, Dr Ernst Borges Marg, Parel, Mumbai, 400012 Maharashtra India; 3 Department of Pathology, Gynaecology Oncology Disease Management Group, Tata Memorial Hospital, Homi Bhabha National Institute, Dr Ernst Borges Marg, Parel, Mumbai, 400012 Maharashtra India; 4 Department of Gynaecology, Aberdeen Royal Infirmary, Foresterhill Estate, Aberdeen, AB25 2ZN Scotland

**Keywords:** Immature teratoma, Germ cell tumor, Growing teratoma syndrome, Cytoreduction

## Abstract

Growing teratoma syndrome (GTS) is a rare clinical condition occurring in women with malignant ovarian germ cell tumors, with paradoxical findings of normal serum tumor markers and enlarging metastatic lesions during the adjuvant chemotherapy and/or follow-up period. The appropriate management is complete surgical resection and is currently the standard of care. We aim to evaluate the surgical and oncological outcomes and follow-up of patients with GTS. A retrospective analysis of clinicopathological outcomes of patients undergoing treatment for GTS between 2014 and 2021 was conducted. Patient demographics and clinical characteristics were calculated using descriptive analysis. Survival estimates for overall survival and disease-free survival are obtained using the Kaplan-Meier method. OS and time to recurrence in GTS between two or more groups are compared using the log rank test. A two-tailed *p* value less than 0.05 was considered statistically significant. A total of 32 cases were included. Around 70% were stage I/II at initial diagnosis, and 60% progressed to GTS during the course of chemotherapy. Surgery was done in 27 cases. The presence of residual disease at surgery for GTS and initial stage were found to be significant predictors for overall survival and time to recurrence in GTS. GTS is a rare entity, and treatment is individualized based on several factors. A complete cytoreduction is the most important predictor for survival in these patients.

## Introduction

The term growing teratoma syndrome (GTS) is a rare clinical condition proposed in 1982 by Logothetis to help describe patients with malignant germ cell tumor treated by surgery and chemotherapy [1]. Growing teratoma syndrome (GTS) is a rare benign condition characterized by paradoxical findings of new lesions or enlarging lesions in the abdomen and pelvis with normal germ cell markers noticed during adjuvant chemotherapy (CT) or follow-up in patients treated for germ cell tumors such as immature teratoma or mixed germ cell tumor. The histology of tumors after surgery reveals exclusive mature teratoma elements. Because testicular germ cell tumors are more frequent than ovarian germ cell tumors, GTS occurs more commonly in male patients with non-seminomatous germ cell tumor (NSGCT) and is rare in female patients with GCT [2].

The etiology of GTS is unclear. The two most quoted hypotheses are—the chemotherapy destroys only the immature malignant cells, leaving the mature benign teratomatous elements, and chemotherapy alters the cell kinetics toward transformation from a totipotent malignant germ cell toward a benign mature teratoma [2, 3]. A third hypothesis offered by Hong et al. proposes an inherent and spontaneous differentiation of malignant cells into benign tissues, as suggested by the experimental murine teratocarcinoma mouse model [4].

The management for GTS in men following testicular cancer has been well described but little is known about GTS in women with ovarian GCTs. The appropriate management is complete surgical resection and is currently the standard of care. Several case series and case reports have been reported in both males and females, the number is increasing due to the increased reporting and newer and better diagnostic tools. The natural history and therapeutic management of GTS in females has been less extensively described in large-scale studies. In the present study, we report our experiences of GTS following treatment of malignant ovarian germ cell tumors and evaluate the surgical outcomes and course of disease during follow-up.

## Objectives

Primary objective: To estimate the overall survival of women with growing teratoma syndrome.

Secondary objective: To estimate progression-free survival and perioperative outcomes in women with growing teratoma syndrome.

## Materials and Methodology

This is a retrospective cohort study of patients with GTS who underwent treatment at a tertiary care center between January 2014 and December 2021. All patients fulfilling the following Logothetis’ criteria of a GTS were included: non-dysgerminoma germ cell tumors with increasing tumor size during or after chemotherapy, normalization of tumor markers, and absence of any non-dysgerminoma germ cell tumor components other than teratoma [1]. Patients with any viable cancer or malignant transformation in the specimens of the residual masses or with non-teratomatous ovarian germ cell tumors were excluded.

All information was retrieved from electronic medical records after the Ethics Committee of Institutional Review Board (IEC No: 3995/IEC II). Clinicodemographic data, treatment details, survival outcomes, and follow-up data were gathered.

### Statistical Analysis

Statistical analyses were performed using SPSS (the Statistical Package for Social Sciences) version 25.0 IBM Corp. Post GTS progression-free survival (PFS) was defined as the time interval between the date of surgery for GTS and the date of recurrence or death due to any cause or the date of last follow-up. Overall survival (OS) was defined as the time interval between the date of surgery for GCT and the date of death due to any cause or the date of last follow-up. Survival estimates for overall survival and progression-free survival were obtained using the Kaplan-Meier method. OS and PFS between two or more groups were compared using the log rank test. A two-tailed *p* value less than 0.05 was considered statistically significant.

## Results

The study included 32 cases of GTS, and the median age of the study cohort was 27.5 years. Around 70% were stage I/II at initial diagnosis, and the most common pathology was immature teratoma in 24 cases (75%). Surgery followed by adjuvant chemotherapy was the most common treatment in 75% of cases. The most common chemotherapy (CT) regimen used was the BEP regimen (bleomycin, etoposide, cisplatin) in 84.4% of cases. Nineteen patients (59.4%) experienced GTS during the course of CT, while 13 patients (40.6%) developed GTS after the completion of treatment.

Most of them presented as multiple site recurrences (78%). Site of disease/metastases is mentioned in Table 1. All cases were discussed in MDT, and complex surgical procedures were performed in 27 (84.4%) cases with surgical input from colorectal and hepatobiliary surgeons. Out of the five cases not operated, three patients declined surgery; one was unfit for surgery in view of poor performance status, gross ascites, and extensive peritoneal disease; and one patient defaulted after surgery was planned. A complete cytoreduction was achieved in 21 of 27 cases (77.8%) while cytoreduction was suboptimal in 6 cases. The reasons for suboptimal cytoreduction were miliary deposits over small bowel serosa in 4 patients, extensive large deposits on liver and spleen along with miliary disease on bowel surface in 1 patient, and large unresectable liver and splenic disease in one patient. Details of surgical procedure are listed in Table 2. The median blood loss was 1800 mL, and the median postoperative stay in the hospital was 6.5 days. Two deaths were observed in the postoperative period. One patient died on postoperative day 5 due to dengue hemorrhagic shock and DIC. The other patient developed DIC, sepsis, and multi-organ failure and died on postoperative day 7.

**Table 1:** Clinicodemographic profile of study population

Characteristic	Number	% (percentage)
Age—mean (median, range) in years	29.7 years (27.5, 17–49)	
Marital status		
Married	12	37.5
Unmarried	20	62.5
Parity		
Nulliparous	20	62.5
>/1 child	12	37.5
FIGO stage		
Stage I/II	23	71.9
Stage III/IV	9	28.1
Primary histopathology		
Immature teratoma	24	75
Mixed teratoma GCT	8	25
Primary treatment planned		
Surgery → chemotherapy	24	75
Chemotherapy → surgery	4	12.5
Chemotherapy → surgery → chemotherapy	2	6.3
Surgery → chemotherapy → surgery → chemotherapy	2	6.3
Timing of occurrence of GTS		
During chemotherapy	19	59.4
After completion of chemotherapy	13	40.6
Interval from chemotherapy to surgery (weeks)	7.2 (0–768)	
Tumor markers before chemotherapy median (IQR)		
AFP (ng/mL)	7.12 (251.128)	
BHCG (IU/L)	0.69 (1.025)	
LDH units/L	218.5 (177.4)	
Tumor markers after chemotherapy		
AFP (ng/mL)	4.12 (7.345)	
BHCG (IU/L)	0.41 (2.655)	
LDH (units/L)	191.5 (36.75)	
Chemotherapy		
BEP regimen (bleomycin, etoposide, cisplatin)	27	84.4
EP (etoposide, cisplatin) f/b BEP	2	6.3
Other regimens	3	9.3
4 BEP → 3 TIP (paclitaxel, ifosfamide, cisplatin)	1	3.1
4 BEP → P + C + ifosfamide (paclitaxel, carboplatin)	1	3.1
2 EP → 1 VbIP (etoposide/vinblastine, ifosfamide, cisplatin)	1	3.1
Site of metastases		
Pelvis	19	
Liver surface	9	
Peritoneum	11	
Nodal	4	
Spleen	2	
Diaphragmatic	8	
Site of metastases		
Single site	13	40.6
Multiple site	19	59.4

**Table 2:** Surgery details

Surgical procedure used for debulking surgery	
Lymphadenectomy	4
Splenectomy	1
Diaphragmatic peritonectomy	8
Liver deposit/subhepatic excision	6
Large bowel resection	6
Large peritonectomies (except diaphragmatic peritoneal resection)	7
Abdominal wall resection	3
Fertility sparing procedure	9
Blood loss: median in milliliters (IQR)	1800 (1925)
Length of hospital stay: median (IQR)	6.5 (3.25)
Postoperative complications	
Ileus	1
Burst abdomen	1
VTE (venous thromboembolism)	1
Death	2
DIC (disseminated intravascular coagulation)	1
Dengue with DIC	1
Residual disease status	
No residual	21 (77.8)
Residual present	6 (22.2)
No surgery	5

Nine cases recurred following initial surgery for GTS. The median time to recurrence of GTS was 15 months (range 1–49 months). Of these nine cases, six underwent repeat surgery/relaparotomy, and four of them had complete cytoreduction. One of these operated patients had GTS recurrence in the form of a high-grade glial tumor and received adjuvant temozolomide. A second GTS recurrence occurred in three of the nine operated cases.

The overall prognosis of GTS is good, and the median OS was 59.8 months with six deaths. At the time of last follow-up, 18 patients were alive without disease, 8 were alive with disease, and 6 had died. The mean interval between the initial diagnosis of GCT and the first occurrence of GTS was 13.4 months (SD 20.88). The presence of residual disease at surgery for GTS and initial stage were found to be significant predictors for overall survival in GTS, and initial stage for PFS (Table 3).

**Table 3:** Variables affecting OS and PFS in GTS

Variable name	PFS	*p* value	OS	*p* value
Residual vs no residual disease	0.603	0.053	0.952	**0.003**
			0.417	
Stage (I/II vs III/IV)	0.737	**0.001**	0.957	**0.003**
			0.486	
No of sites of metastasis	0.699	0.3	0.917	0.408
			0.779	
IT vs mixed GCT	0.547	0.696	0.825	0.72
			0.875	
GTS during CT/after CT	0.668	0.446	0.923	0.181
			0.765	

## Discussion

### Summary of Main Results

The study included 32 cases of GTS and the most common pathology was immature teratoma. Multiple site recurrences were more common, and surgery was feasible in 27 of them. Complete cytoreduction was achieved in 21 of the 27 cases, and two postoperative deaths were noted. GTS has a good overall prognosis, and the presence of residual disease and initial stage are significant predictors of overall survival and initial stage for PFS.

### Results in the Context of Published Literature

The growing teratoma syndrome was first described in 1977 by DiSaia et al., who reported three women who developed rapidly growing solid masses during treatment with adjuvant chemotherapy for malignant ovarian IT [4]. Subsequently, in 1982, Logothetis et al. described this clinical entity in males after chemotherapy for metastatic mixed germ cell tumors of the testis and termed this growing teratoma syndrome [1]. It is much more common in (non-seminomatous) testicular tumors with an incidence of 1.9% to 7.6%, whereas it is rare in females [5].

The pathogenesis of GTS is unknown; however, two theories have been put forward: (1) chemotherapy may induce the differentiation of malignant cells into mature components; (2) chemotherapy may induce the selective destruction of malignant components, leaving mature components that are resistant to chemotherapy [4, 6]. This phenomenon of rapid growth of benign tumor mass has also been called chemotherapeutic retroconversion, since it is believed to result from chemotherapy-induced conversion of immature gonadal germ cell tumor to a benign mature form. Given the fact that GTS is a benign condition and has an indolent course, the growth of the mature implants does not lead to early symptoms, and they appear when the implants are big enough to produce pressure, pain, or abdominal distension or cause compression of surrounding organs.

The natural progression of GTS is variable and unpredictable. They vary in their growth rate and may appear either during the early course of chemotherapy or as late as 12 years post-chemotherapy. In the present study, nearly half of the GTS cases (19 GTS cases) occurred during chemotherapy, while the others developed GTS after chemotherapy. Early recognition is crucial, as it obviates the need for unnecessary adjuvant therapy, as mature teratoma is refractory to chemotherapy and radiation therapy. Complete surgical resection is the cornerstone for treatment when possible and provides confirmation of the diagnosis of GTS, relief of pressure and mass effect, and exclusion of recurrence of malignancy [3, 5]. Complete resection should be the goal of surgery since incomplete resections have been associated with high recurrence rates. This is also shown in our study, where the presence of residual disease was associated with poor OS and PFS. Another study by Paffenholz et al. also revealed the importance of complete cytoreduction in GTS [7]. Factors affecting complete cytoreduction may include the fitness of the patient to undergo extensive surgery, intraoperative complications such as severe/massive hemorrhage, DIC, and disease being present in crucial areas precluding complete cytoreduction.

One of the primary objectives of this study was to help define the operative management of peritoneal GTS. The surgical procedures required for GTS are usually similar to those performed at the time of debulking surgery for epithelial ovarian cancers, and complete cytoreduction is required for good survival outcomes. However, compared with the surgical procedures required for epithelial ovarian cancer, one notable exception exists: if technically feasible, fertility-sparing surgery with preservation of the uterus and the remaining ovary can be proposed despite peritoneal spread (remembering that all patients treated for GTS had a history of oophorectomy or salpingo-oophorectomy for the initial treatment of the ovarian tumor) [5, 7]. Sometimes, this conservative approach is not technically feasible because the remaining ovary is involved in the pelvic masses of GTS. Nevertheless, when the remaining ovary has small surface lesion, it is better to leave the remaining ovary in place even if a CC0 resection is not subsequently obtained rather than to remove it and impair the further fertility results. Fertility preserving surgery was possible in nine of these operated cases and is suggested for patients with GTS as spontaneous pregnancy is possible.

Multiple recurrences are frequent and may require subsequent surgeries. Shigeta et al. report a recurrence rate of 13% in 7 of 55 reported cases [5, 7]. In our cohort, nine patients (9 out of 27 operated cases—33.3%) experienced recurrences of GTS as late as 49 months from their first diagnosis of GTS. Out of these 9 recurrences, six had complete cytoreduction in the first GTS surgery. The mean time to GTS recurrence was 22.7 months. Six out of these were treated by re-surgery. The risk of recurrence may be related to incomplete surgical resection of GTS. Five of these nine recurrences had initial FIGO stage III/IV at the time of diagnosis. In our study, advanced staged disease at the initial diagnosis of GCT and incomplete resection were associated with GTS recurrences.

There is also a low risk of malignant transformation, which was reported to be 3% to 5%. Growing teratoma syndrome has been reported to have the potential to transform into sarcoma, adenocarcinoma, primitive neuroectodermal tumor, spindle cell neoplasm, and carcinoid tumors, as late as 22 years after the diagnosis of GTS [8, 9]. In our study, one patient experienced malignant transformation of GTS in the form of a high-grade glial tumor, which was surgically resected, and adjuvant chemotherapy was administered.

Most of our patients in the present series required a multidisciplinary team approach, including gastrointestinal and hepatobiliary surgeons, due to the complexity of the surgery. Fourteen patients had blood loss of at least 1000 mL during surgery. Patients with GTS should ideally be transferred to a high-volume specialized center with access to multidisciplinary surgical care. The liver was the most common site of extra-pelvic recurrence in our study. Most of the GTS spread peritoneally. However, during surgery, we found that such metastasis was confined to the surface of the liver and was characterized by a thick fibrous capsule in the liver without infiltrating the liver parenchyma. Therefore, it gives a false impression of liver metastasis owing to a compressive growth pattern. As GTS consists of mature elements, it lacks the ability to metastasize or to invade the surrounding tissues. Therefore, surgical resection of false liver metastasis is feasible.

The overall prognosis of GTS is good, and the 5-year survival rate is reported to be 89%–90% [3, 5]. The 5-year OS probability was 83.4%, and PFS was 57.2%. In our study, the median OS was 59.8 months, with six deaths noted in the study population. At the time of the last follow-up, 18 patients were alive without disease, 8 were alive with disease, and 6 had died. Residual disease and the presence of GP at initial surgery are independent risk factors for GTS development. The presence of residual disease is a risk factor for recurrence not only in initial surgery but also at GTS recurrence.

The literature reports that most cases of GTS are followed by immature teratoma [3, 5, 7]. In the present study, the diagnosis of GTS cases (75%) is observed after the treatment of immature teratoma, but they can still be infrequently diagnosed after other histologies of germ cell tumors like embryonal carcinoma and mixed germ cell tumors. Immature teratomas are germ cell neoplasms composed of tissue derived from the three germ cell layers. These tumors are graded based on the percentage of immature elements and the presence and quantity of the neuroepithelial components (percentage of undifferentiated elements) (grade 1 to 3) [10]. In the case of GTS diagnosis, the study of the implants reveals benign mature teratomatous elements (grade 0) with predominantly ectodermal neurogenic elements including glial tissue (“gliomatosis peritonei”).

Several risk factors for the development of GTS have been reported. Few of them include incomplete resection at initial surgery, initial tumor containing immature teratoma elements, histological grades 2 and 3, surgical stages III and IV, and no decrease in initial tumor bulk after treatment with adjuvant chemotherapy [3, 5, 7, 11]. Zagame et al. described the involvement of the peritoneum and the presence of a neuroectodermal tumor as significant risk factors for GTS development [11].

The GTS represents a rare clinical phenomenon that should be kept in mind in case of a young patient with ovarian germ cell tumors presenting with recurrent pelvic and abdominal metastatic lesions with normal serum tumor markers following primary surgical cytoreduction/chemotherapy. Prolonged surveillance is indicated, and follow-up with tumor markers and imaging on a frequent basis is recommended. However, due to the rarity of ovarian immature teratoma and GTS, the etiology and clinical behavior of this syndrome are still poorly understood. A multicentric cohort-based retrospective tumor data collection/registry may help us further in the management of these rare tumors.

## Conclusion

GTS is a rare entity and treatment is individualized based on several factors. A complete cytoreduction is the most important predictor for survival in these patients.
